# Severe Life Threatening Rectal Bleed After Prostate Biopsy Requiring Angiographic Therapy: A Case Report

**DOI:** 10.4021/gr237w

**Published:** 2010-09-20

**Authors:** Ariyo Ihimoyan, Raghu Maddela, Nirisha Kalakada, Kavitha Kumbum

**Affiliations:** a Division of Gastroenterology, Bronx Lebanon Hospital Center, Bronx, New York 10457, USA; b Department of Medicine, Bronx Lebanon Hospital Center, Bronx, New York 10457, USA

**Keywords:** Rectal bleeding, TRUS-guided prostate biopsy

## Abstract

Rectal bleeding is often seen in patients who undergo transrectal ultrasound-guided prostate biopsy. It is usually mild and stops spontaneously. We report what we believe is the first case of life threatening rectal bleeding following this procedure which was successfully treated with angiographic embolization. An endoscopic injection of epinephrine done on admission achieved only temporary hemostasis and the patient developed a second episode of massive rectal bleeding that warranted angiographic treatment. We present this case in detail and review other alternatives for treating fulminant rectal bleed which is a consequence of this procedure.

## Introduction

Transrectal ultrasound (TRUS)-guided prostate biopsy is the gold standard for diagnosing prostate cancer [1]. It is generally a safe procedure with minimal gastrointestinal and genitourinary morbidity and is commonly performed as an outpatient procedure.

After this procedure, mild rectal bleeding may occur in 0-25% of patients [2, 3]. Other complications include self limiting hematuria, hematospermia and urinary tract infection, most of which resolve with conservative therapy [4, 5]. Severe rectal bleeding is usually managed by the urologist with rectal tamponade as the initial and simplest conservative method or when necessary, as balloon compression by means of a transrectally inserted catheter [5]. Endoscopic intervention with injection of adrenaline and sclerosing solutions, thermocoagulation and band ligation have been used successfully in some cases [6-8].

Herein, we report a case of severe hematochezia following TRUS-guided prostate biopsy that was eventually treated by angiographic embolization.

## Case Report

A 78-year-old African American man with medical history of hypertension was found to have an enlarged prostate associated with complaints of urinary hesitancy by his primary care physician. He was referred to urology and later underwent a TRUS-guided prostate biopsy. A few hours after the procedure on his way home, he had a syncopal episode and was transferred to the ER.

He was found to have copious amount of fresh blood clots per rectum with significant hemodynamic instability. He was diaphoretic with a blood pressure of 80/30 mmHg and a pulse rate 100 - 120/min. Patient was aggressively resuscitated with fluid and received a total of 5 units of packed red blood cells. He was transferred immediately to the intensive care unit.

**Figure 1:**
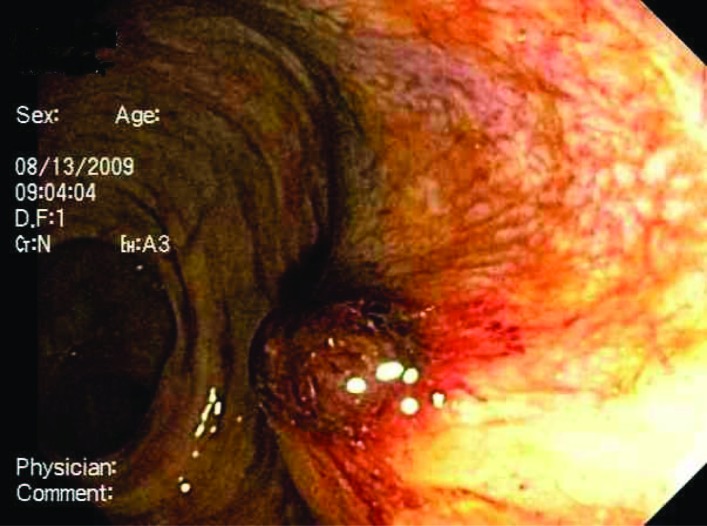
Raised erythematous mucosal lesion in the rectum, most likely the site of prostate biopsy. The lesion was injected with 10 ml of epinephrine.

A flexible sigmoidoscopy performed revealed multiple diverticulosis in the left colon and a raised non-bleeding erythematous mucosal lesion in the rectum (most likely the site of prostate biopsy). The lesion was injected with 10cc of 1:10000 epinephrines (Fig. 1). We planned a full colonoscopy at later date when patient was more stable.

While in the unit the patient had no further rectal bleed and his hematocrit remained stable (between 30-35%) and as such was transferred to the medical floor.

**Figure 2:**
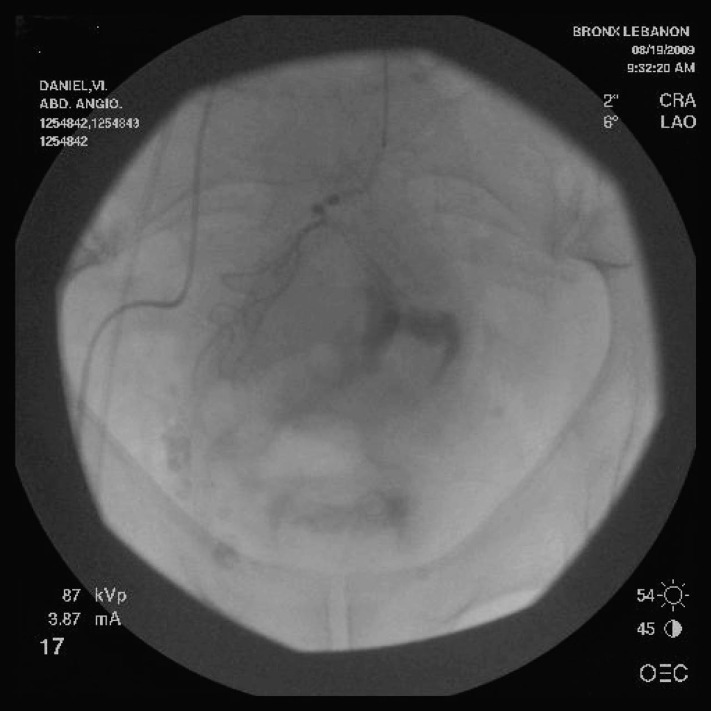
Selective inferior mesenteric angiogram showing a brisk blush in distal portion of the superior hemorrhoidal artery.

Five days later, on the day prior to planned discharge he developed multiple episodes of massive rectal bleeding with a drop in hematocrit to 25% and drop in systolic blood pressure of 60 mmHg. A central line was placed and the patient received transfusion with 4 units of packed red blood cells and was transferred back to the intensive care unit. As he continued to bleed massively with hemodynamic instability we opted for an immediate angiography rather than a colonoscopy. A complete mesenteric angiography was performed (Fig. 2).

The selective inferior mesenteric artery angiogram revealed a brisk blush in distal portion of the superior hemorrhoidal artery and collection of contents in the rectum. Gel foam embolization of the superior hemorrhoidal artery with successful hemostasis.

**Figure 3:**
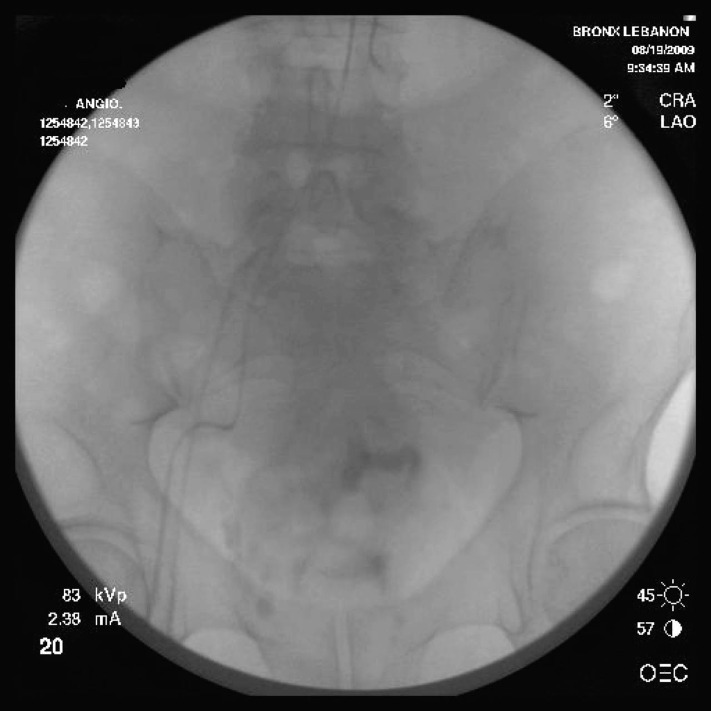
Follow up selective inferior mesenteric angiogram after successful embolization. There is filling of the proximal superior mesenteric artery without filling in distal superior hemorrhoidal artery.

A follow-up selective inferior mesenteric artery angiogram revealed filling of proximal superior mesenteric artery without any filling in distal superior hemorrhoidal artery indicating an effective superior hemorrhoidal artery thrombosis (Fig. 3).

Following the successful angiography and embolization, the rectal bleeding stopped. The patient was monitored in the ICU for another 48 h during which his hematocrit was stable and developed no further bleeding. He was discharged in a stable condition.

## Discussion

After thorough review of existing literature we believe our case is the first reported case of severe life threatening rectal bleeding following TRUS-guided prostate biopsy effectively managed by angiographic therapy. Screening for prostate cancer has become an important issue in recent years. TRUS-guided prostate biopsy is one of the most common urological procedures to diagnose prostate cancer. Most of the hemorrhagic complications are minor and stop in a few days [8]. About seven cases of massive life threatening rectal bleeding following needle biopsy of the prostate have been reported in literature [6-13].

These patients required blood transfusion and in most of the cases hemostasis was achieved with rectal balloon tamponade, endoscopic intervention with injection of adrenaline or slerosants, thermocoagulation, and band ligation. Following most cases of post biopsy rectal bleeding when digital pressure and rectal packing failed, balloon tamponade with inflated foley catheter was the most frequently reported method used to control bleeding [14]. The condom balloon tamponade was first reported by Murat Gonen et al and was preferred to the use of foley catheter due to a more effective pressure on both rectal wall and prostate [9]. Panagois Katsinelos reported a case that was effectively treated with endoclipping [13].

In our case, at the time of endoscopy the hemorrhagic rectal lesion at the site of biopsy had stopped bleeding. Despite injection of epinephrine, a second episode of massive bleeding occurred. A repeat endoscopy could not be performed as the patient was bleeding profusely so we opted for angiography. Risk factors for post biopsy rectal bleeding have not yet been fully elucidated in specifically designed studies.

One study suggested an association between the amount of rectal bleeding to the total number of core biopsies performed [15]. Our patient had only one core biopsy. Rietbergen et al reported a slightly increasing trend with increasing age although this was not statistically significant [16]. Post biopsy rectal bleeding is thought to occur because the biopsy area consists of richly vascularized region that includes the middle and inferior rectal arteries and the inferior vesicular artery. The rectal vein plexus is also located in this region and can increase in size in case of hemorrhoids. Sheikh et al recommend transperineal removal of tissue rather than a transrectal biopsy in patients with severe hemorrhoids [17].

In conclusion, prostate biopsy is the most important step in diagnosing prostate cancer, but physicians should be aware that life threatening rectal bleeding may occur following this procedure. Our patient represents the first known case of life threatening rectal bleeding following TRUS- guided prostate biopsy managed effectively by angiographic therapy.
